# NICU Language, Everyday Ethics, and Giving Better News: Optimizing Discussions about Disability with Families

**DOI:** 10.3390/children11020242

**Published:** 2024-02-15

**Authors:** Paige Terrien Church, Maya Dahan, Amy Rule, Annie Janvier, Jane E. Stewart, John S. Maypole, Darcy Fehlings, Jonathan S. Litt, Rudaina Banihani

**Affiliations:** 1 Beth Israel Deaconess Medical Center, Boston, MA 02215, USA; jstewart@bidmc.harvard.edu (J.E.S.);; 2 Sunnybrook Health Sciences Centre, Toronto, ON M4N 3M5, Canadarudaina.banihani@sunnybrook.ca (R.B.); 3 Children’s Healthcare of Atlanta, Atlanta, GA 30342, USA; 4 CHU Sainte-Justine, Montreal, QC H3T 1C5, Canada; anniejanvier@hotmail.com; 5 Boston Medical Center, Boston, MA 02118, USA; 6 Holland Bloorview Kids Rehabilitation Hospital, Toronto, ON M4G 1R8, Canada; dfehlings@hollandbloorview.ca

**Keywords:** disability, ableism, neurodevelopmental outcomes, NICU, ethics

## Abstract

The Neonatal Intensive Care Unit (NICU) has a language and culture that is its own. For professionals, it is a place of intense and constant attention to microdetails and cautious optimism. For parents, it is a foreign place with a new and unique language and culture. It is also the setting in which they are introduced to their child and parenthood for this child. This combination has been referred to as an emotional cauldron. The neonatal ethics literature mainly examines complex ethical dilemmas about withholding/drawing life sustaining interventions for fragile children. Rarely are everyday ethics or mundane ethics discussed. Microethics describe the mundane, discrete moments that occur between patients/families and clinicians. A key piece of these microethics is the language used to discuss patient care. Perception of prognoses, particularly around long-term neurodevelopmental outcome, is shaped with the language used. Despite this, clinicians in the NICU often have no specific training in the long-term neurodevelopment outcomes that they discuss. This paper focuses on the microethics of language used to discuss long-term neurodevelopmental outcomes, the developmental neuroscience behind language processing, and offers recommendations for more accurate and improved communication around long-term outcomes with families with critically ill neonates.

## 1. Introduction

*Case: Lily is a 3-day old 24-week infant with a first head ultrasound finding of a large venous infarction on the right. She has been otherwise age-appropriate in her care needs, with minimal ventilator settings, parenteral nutrition and no signs of sepsis. She is the first pregnancy for her parents. Her delivery was precipitous and the family did not have the opportunity for antenatal counselling prior to her delivery. Her parents have been consistently at her bedside and have been updated on her progress consistently.*

*At the family update regarding the head ultrasound, with the attending and fellow as well as bedside nurse, the results are conveyed and possible outcomes reviewed. “We did our first head ultrasound on Lily and it demonstrated a large area of bleeding in her brain. This can be called a Grade IV bleed and is associated with impairments in life, like cerebral palsy which in this case could be severe. It also may affect her cognitive abilities and we cannot yet be sure of her vision or hearing abilities. Some parents who hear this news choose to redirect care or provide comfort care, others choose to continue to provide care, regardless of what impairments she may encounter.” Lily’s parents ask to hold her. They state that they have many questions but for now, want to hold their daughter.*

“Language is as vital to the physician’s art as the stethoscope or scalpel. Of all the words a doctor uses, the name he gives the illness has the greatest weight” [1]. This quote from Jerome Groopman underscores the power of the spoken word as it relates to a diagnosis and the implications of a diagnosis. The study of language has increasingly been of interest, for its power to convey information to patients about their health, but also for its potential ethical implication. Microethics has been described as every ethical interaction that occurs between a clinician and patient [2]. Language, particularly the words spoken, while not all encompassing, may play a critical role in creating the microethical climate. In the Neonatal Intensive Care Unit (NICU), critical information is shared with parents about their baby’s medical condition but also the family’s and baby’s future. The implications of the words spoken, therefore, can have profound family-altering impacts [3]. This paper will review (1) the concept of microethics, (2) the process by which humans derive meaning from words, and (3) how the spoken words can frame understanding, leading to lasting impacts for families. Finally, it will provide recommendations to shift toward a value-neutral, objective, and functional description of neonatal outcomes in an effort to create a better microethical climate for families.

## 2. Microethics in the NICU and Ableism in Medicine

Historically, bioethics has described those seismic moments in a patient and family’s care that involve key decisions around life-sustaining care or palliative care. Indeed, the vast majority of the literature and research in neonatal ethics is about complicated dilemmas associated with life and death. These cases are significant and, yet, uncommon [4]. Macroethics describes ethical decisions at a health care-utilization and resource level [4]. In contrast, microethics describes the many aspects of everyday care that have ethical aspects. This concept was first described by Paul Kamesaroff in 1995. “The vast majority of medical decisions are taken in an ethical environment in the absence of any dilemma. They are made with an organic, ongoing relationship, in the spirit of open dialogue between patient and doctor” [2]. In identifying this concept of microethics, those common, innumerable moments between clinician and patient, Dr. Kamesaroff sought to “reveal the structure and dynamics in the clinical interaction and, in particular, to explicate the actual processes involved in clinical decision making” [2]. In the NICU setting, every interaction with families, from the antenatal consultation, to rounds and bedside updates, to discharge planning, all are laden with microethics.

While there are many characteristics of a clinician–patient interaction that can be explored through microethics, language is central to communication and consequently plays a significant role [5]. It is the language and the words spoken in the NICU that are of particular interest to this paper. “Communication during clinical encounters can cause great harm or healing” [4]. In the NICU, clinicians bring critical and novel information to parents about their baby and their future. Parents are then asked to make decisions that will forever shape their family. Both clinicians and parents bring to these discussions their own set of beliefs, values, and biases [5]. There are many different potential cognitive biases that have been described as important factors in the NICU environment. For the purpose of this paper, focusing on words spoken in the novel environment of the NICU, the cognitive bias called anchoring bias is particularly relevant. Anchoring bias describes that tendency to assign greater value to the first piece of information given. For parents in the NICU, learning about possible long-term neurodevelopmental outcomes, the words used anchor their understanding; they are the first impression. Given this tendency, the ‘first’ words chosen are pivotal.

The backdrop for discussions around neurodevelopmental outcome is the longstanding history of ableism in medicine and in society [6,7]. Ableism describes the projection of a belief that there is a ‘normal’ corporeal standard and that differences from this standard are regarded as deficits, impairments, and ‘abnormal’ [8]. Recently, a survey demonstrated improved societal perspectives around race, gender, and sexuality, but an ongoing persistent bias concerning disability [9]. The medical community plays a unique role within disability as it often identifies and participates in the management of individuals living with disability, providing a more nuanced perspective on life with disability. Historically, however, the medical system has contributed to the perpetuation of ableism, articulating a preference for conditions that can be ‘fixed’ over those that persist [10] and a preference to care for those who are not disabled, viewing disability as associated with a lesser quality of life [6]. It is deeply entrenched in the system of medical care and, as such, it would be impossible for parents or clinicians to be immune to this bias.

## 3. Relational Frame Theory (Learning by Experience) and Heuristics

The reason that the words clinicians use to describe neurodevelopmental outcomes become sources of anchoring bias is rooted in how the brain derives meaning in words and how this can impact decision making. Most organisms learn and adapt by experience. Touch a hot plate, get burned, and adapt not to repeat that experience. This learning is automatic and reflexive. Humans, however, also have the capacity to find meaning between stimuli and the resulting relationships that are reinforced, and it is those associations that persist. The relational frame theory describes this specific human developmental pattern for language processing and learning [11,12,13]. The brain is driven to create meanings from words. This process is subconscious, learned in early development and describes early vocabulary and literacy development [11,12,13]. A small child learns that the spoken and then the written word “dog” equates to the furry creature that they see in a book or in real life. That association is taught. The brain, however, also then associates that the furry creature with a tail and nose is “dog”. Instruction is unidirectional but the brain develops an automatic bidirectional association between the word and the animal [12]. This tendency of the brain to make associations is critical for language and cognitive learning. Add an association such as “bite” to this equation and the individual learns that dog and bite are also associated, despite the fact that not all dogs bite and not all bites are from dogs. The more one knows about bites coming from different animals and the more one knows about dogs beyond bites, the more bites and dogs become distinct entities. However, the least one knows about dogs and bites, the two remain closely related. Words shape reality and understanding, particularly when learning new words [13].

At a baseline, the human brain is not only driven to derive relationships between words, but also to act based on understanding of those words. Generally, the human brain operates in one of two ways: quickly, relying on intuition and reflex; or to think through more laboriously, dissecting the data and coming to a conclusion [14]. These two ways of decision making have been referred to as system one (fast) and system two (slow) thinking [14]. System one thinking is described as an ‘autopilot’, a gut instinct. It derives relationships, finds patterns, and is thus error-prone. System two thinking is a conscious thought process, addressing new or complex tasks with reasoning and logic, and generally is more reliable. To make decisions quickly, however, which is the predominant way the brain makes decisions, the brain relies on shortcuts and system one thinking [14].

The processes by which the brain makes these mental short cuts are called heuristics and they provide efficient problem solving and decision making [14,15]. Heuristics occur in the subconscious and their efficiency is based largely on assumptions, prior experience, and cognitive biases [14,15]. In the NICU setting, both the clinician and the parent are operating with their own individual set of understandings and biases, and thus heuristics [5]. For the clinician, it is demonstrated in the decisions for how a planned message is crafted, delivered, the words selected and received. For the parent, it is their understanding of the words spoken and their derived meaning from the words used. Much of this processing occurs at the subconscious level, using system one thinking and relying on fast associations.

## 4. NICU: The Emotional Cauldron

**Figure 1:**
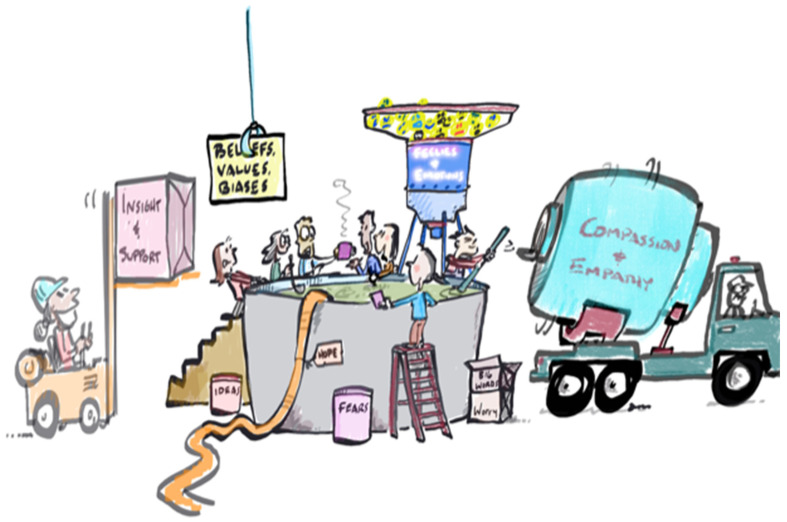
The emotional cauldron.

The NICU is a new place for most parents. It is in this foreign place that new words find meaning, new frames of reference exist, amidst intense anxiety and fear as well as tremendous hope [16,17]. For clinicians, the NICU is a familiar place of hyperattention to every detail, skilled monitoring, collaboration, and cautious optimism. It is in this new and technical environment, under the scrutiny of the constant surveillance of NICU clinicians, that families are established, meeting their baby for the first time. There is a collision of previous hopes and expectations with the demands of the new setting. This jumbled concoction of scrutiny, disparate expectations, fear, and hope has been referred to as the ‘emotional cauldron’ [18] of the NICU (see Figure 1).

Given how meanings are linked to words as they are first learned, the prevalence of anchoring bias, and combined with the tendency for heuristics, the nature of discussions in this emotional cauldron of the NICU takes on tremendous importance and yet, there are few guidelines for clinicians on how to convey these important conversations. Clinicians are driven to provide value-neutral guidance that can be individualized to their patients. For most, however, with little follow up experience, they must rely on those outcomes described in the literature, which have been framed in value-laden, ableist language [19,20]. Traditionally, the developmental outcome of babies cared for in the NICU has been reflected by the measurement of cognitive and motor skills, as well as vision or hearing capacity, collectively referred to as neurodevelopmental outcome [19,20]. Demonstration of a significant challenge in one or more of these domains has been reflected as the presence of a neurodevelopmental impairment (NDI) [19]. After an NICU hospitalization, children are categorized as having one of four possible outcomes: mild, moderate, severe NDI, or no disability. Over decades of data, several flaws have been identified in this practice [19,20]. First, and foremost, these outcome data are limited, short term, and have not demonstrated a link to future function [19]. Second, most clinicians in the NICU have little or no training or formal experience of caring for children after discharge from the NICU, making them reliant on the data published for discussions on neurodevelopmental outcome. Third, the outcomes long-reflected as measures of the future for families and individuals receiving care in the NICU have not included input from families and individuals on those outcomes that may matter to them [20,21,22].

Parents who have infants admitted to the NICU are inundated with new concepts from the antenatal consultation, during the NICU stay, and in their follow up visits [17]. Many parents have never heard or considered prematurity, the equipment used in the NICU, the disciplines making up the team, the medical conditions listed, or the words used to describe developmental outcomes. These are often new concepts introduced for the first time by the medical team. Just as the small child learnt that dogs bite, when the two were presented together, parents may learn that there is a “risk” of cerebral palsy, which is a “poor” outcome, as these words are often presented together in the literature. This phenomenon is especially potent when the learner knows nothing or little about the topic, just as a child only being told dogs bite and nothing about dogs or bites in other contexts. The NICU, with the associated stress and uncertainty, is fertile ground for distorted derived meanings in words spoken.

## 5. Recommendations

The relational frame theory and bidirectional learning mean that how concepts are introduced to families in the NICU creates their frame of reference moving forward [11,12,13]. With this in mind, is it possible to offer counselling with awareness of language, the tendency for ableist bias, and heuristics? While it may not be possible to do so completely, important recommendations have emerged (Table 1).

## 6. Conclusions

With the above awareness of the brain deriving meaning in relationships between words [11,12], anchoring bias, the ubiquity of ableism [8,9] and heuristics [14], every word spoken in the emotional cauldron that is the NICU has profound implications. We have focused on this specific case of an important conversation but these recommendations apply to any communication with a family about neurodevelopmental outcomes. We now return to our case in the NICU, reflecting on the recommendations.

### Case

*For the discussion, to revise it: “We did our first head ultrasound on Lily and it demonstrated a large area of bleeding in her brain. This bleeding is in area of her brain that may affect how she will move, particularly on her left side. This may show itself with a preference for her right hand and her care team would follow her closely for any early emergence of a preference as we have simple exercises that can help with this, particularly if found early. She will likely walk and may have a gait that is affected by a limp or difference in how easily the right and left legs move. With her prematurity, we cannot be sure what impact, if any, it will have on her learning. What we do know is that there are many strategies to optimize her learning.*

*Most families hearing this news think about important things like:*

- *Will my daughter have a future? The answer is yes, quite possibly.*
- *Will my daughter have friends? The answer is yes, we think she can have friends and will enjoy these friendships.*
- *Will Lucy be able to do sports or have fitness? The answer is yes, and some sports may need to be adapted for any additional motor needs she has but possible.*
- *Will my daughter have fun? The answer is yes, fun is still very much a possibility for her.*
- *Will my daughter have functioning, such as being able to be at school, get an education and be independent as she grows? The answer is yes, and you and her and her team will watch her grow, identify her needs and introduce interventions at home and then at school. With her, and you as her parents, the team will continue to evolve with the goal that she can have a fulfilling job, independence.*
- *Will my daughter have a family? The answer is yes, she has the possibility of meeting someone she loves and starting a family.*

*Some families hear this news and share their feelings that for their family, making decisions that result in their child surviving with long-term challenges is not an outcome that they are comfortable with or capable of handling. That is important to consider and we encourage you to have important, honest conversations with each other to make important care decisions, such as deciding to redirect her care to that which will continue to keep her comfortable but not sustain her life artificially, recognizing that this will make it a shorter life. Other families articulate a desire to continue with her care without any changes and to revisit the discussion should new information come forward.”*

The NICU is an ‘emotional cauldron’ [18] for all involved. The work and care are precise. While our care and knowledge in the NICU have evolved, our language and microethical considerations around ableism have been neglected, particularly as it relates to the discussion and measurement of neurodevelopmental outcomes. This paper serves to offer strategies to move the discussion and measurement of neurodevelopmental outcomes forward because “…. Of all the words a doctor uses, the name he (or she first) gives the (condition) has the greatest weight” [1].

## Figures and Tables

**Table 1:** Recommendations, rationale, and examples.

Recommendation	Rationale	Example
1. Initiate a bias break with the NICU team before important discussions with families.	Bias breaks provide a safe place to acknowledge potential sources of bias as they relate to the baby or family. By explicitly acknowledging potential sources of bias, it allows for greater awareness, opportunities for education, and prioritizes the goal of addressing possible implicit and explicit bias [23].	“This is an important conversation we are about to have with this family. Let us take an opportunity to discuss any sources of bias that may exist around this family and/or this baby which may interfere with our goal of delivering balanced, individualized care that is free of bias as much as possible”.
2. Divest discussion about neurodevelopment from medical reference points.	Development is a fluid process with tremendous variability, not a binary either or event [24].	Avoid using words like ‘normal’ and ‘abnormal’.
3. Describe outcomes as they relate to families, focusing on function rather than medical diagnoses.	Functional descriptions of neurodevelopmental outcomes are more relevant than diagnoses and medical terminology [19,20,21,22,25]. Descriptions of how a child will move and how a child will eat are highly relevant to families. Important universal goals for any family have been described by Rosenbaum and Gorter as the ‘F’ words [25]. Focusing on the ‘F’ words as important measures of outcome allows a family to truly comprehend the future possibilities for their baby.	“For families learning about possible outcomes, we can talk about the medical diagnosis or names used to describe these outcomes such as cerebral palsy. Most families, however, want to learn about how being in the NICU will impact their child’s possibility of having friends, fun, fitness, future, family, and function. We are going to talk about all of this, focusing on your child as it relates to these important outcomes”.
4. Use objective, value-neutral language to describe outcomes to avoid introduction of ableist bias.	Objective, value-neutral language is recommended to describe neurodevelopmental outcomes. This allows for families to derive their own meaning and to make decisions based on their values, rather than taking on medical biases [26,27,28,29,30].	Replace: Substitute word “Risk” → “Possibility” “Severe” → “Significant” “Bad news” → “Important news” “Poor” → “Significant”
5. Future research needs radical revision from measurement of short-term medically derived outcomes to those outcomes that parents and individuals deem important.	Families have articulated that what has been measured historically in the outcome literature is not consistent with that which parents experience as important outcomes [21]. “We understand that clinicians need to examine outcomes and classify children: they can either have serious, moderate, or mild disability or maybe classified as ‘typical’. These categories were created by physicians. If we were asked to classify our children as disabled or not disabled, then doctors would have different categories. Why is hyperactivity a mild disability and cerebral palsy a major one? [31]”.	Consideration for future outcome measurements, using the ‘F’ words as guidance include the impact of the NICU experience on the following: feeding; sleeping; behavioral regulation; function in the classroom; social skills; mental health; health implications; adult outcomes.

## Data Availability

No new data were created or analyzed in this study. Data sharing is not applicable to this article.
